# Chitosan Nanoparticles: Shedding Light on Immunotoxicity and Hemocompatibility

**DOI:** 10.3389/fbioe.2020.00100

**Published:** 2020-02-21

**Authors:** Sandra Jesus, Ana Patrícia Marques, Alana Duarte, Edna Soares, João Panão Costa, Mariana Colaço, Mélanie Schmutz, Claudia Som, Gerrit Borchard, Peter Wick, Olga Borges

**Affiliations:** ^1^Center for Neuroscience and Cell Biology, University of Coimbra, Coimbra, Portugal; ^2^Faculty of Pharmacy, University of Coimbra, Coimbra, Portugal; ^3^Laboratory for Technology and Society, Empa Swiss Laboratories for Materials Science and Technology, St. Gallen, Switzerland; ^4^Institute of Pharmaceutical Sciences of Western Switzerland, University of Geneva, Geneva, Switzerland; ^5^Laboratory for Particles-Biology Interactions, Empa Swiss Laboratories for Materials Science and Technology, St. Gallen, Switzerland

**Keywords:** chitosan nanoparticle, immunotoxicity, hemocompatibility, deacetylation degree, endotoxin-free, inflammation, reactive oxygen species, PBMCs

## Abstract

Nanoparticles (NPs) assumed an important role in the area of drug delivery. Despite the number of studies including NPs are growing over the last years, their side effects on the immune system are often ignored or omitted. One of the most studied polymers in the nano based drug delivery system field is chitosan (Chit). In the scientific literature, although the physicochemical properties [molecular weight (MW) or deacetylation degree (DDA)] of the chitosan, endotoxin contamination and appropriate testing controls are rarely reported, they can strongly influence immunotoxicity results. The present work aimed to study the immunotoxicity of NPs produced with different DDA and MW Chit polymers and to benchmark it against the polymer itself. Chit NPs were prepared based on the ionic gelation of Chit with sodium tripolyphosphate (TPP). This method allowed the production of two different NPs: Chit 80% NPs (80% DDA) and Chit 93% NPs (93% DDA). In general, we found greater reduction in cell viability induced by Chit NPs than the respective Chit polymers when tested *in vitro* using human peripheral blood monocytes (PBMCs) or RAW 264.7 cell line. In addition, Chit 80% NPs were more cytotoxic for PBMCs, increased reactive oxygen species (ROS) production (above 156 μg/mL) in the RAW 264.7 cell line and interfered with the intrinsic pathway of coagulation (at 1 mg/mL) when compared to Chit 93% NPs. On the other hand, only Chit 93% NPs induced platelet aggregation (at 2 mg/mL). Although Chit NPs and Chit polymers did not stimulate the nitric oxide (NO) production in RAW 264.7 cells, they induced a decrease in lipopolysaccharide (LPS)-induced NO production at all tested concentrations. None of Chit NPs and polymers caused hemolysis, nor induced PBMCs to secrete TNF-α and IL-6 cytokines. From the obtained results we concluded that the DDA of the Chit polymer and the size of Chit NPs influence the *in vitro* immunotoxicity results. As the NPs are more cytotoxic than the corresponding polymers, one should be careful in the extrapolation of trends from the polymer to the NPs, and in the comparisons among delivery systems prepared with different DDA chitosans.

## Introduction

Studies have shown that nanoparticles (NPs) can interact with different components of the immune system, resulting in immunosuppression and in immunostimulation (Dobrovolskaia and McNeil, 2007). Although these interactions can be purposeful and desirable in increasing the efficacy of vaccines, cancer immunotherapy or immunotherapies for autoimmune diseases, they can also be unexpected and undesirable, causing hypersensitivity reactions, anaphylaxis, coagulopathies and body defense decrease (Dobrovolskaia and McNeil, 2007).

Chitosan (Chit) is the common name given to a family of natural polysaccharide polymers obtained from the deacetylation of chitin. Chit is a cationic polymer, considered non-toxic, biodegradable and biocompatible and is therefore extensively investigated in nanobiomedical research (Ali and Ahmed, 2018). Chit has been granted FDA Generally Recognized As Safe (GRAS) designation (GRN n° 73, 170, 397 and 443) and is widely used in dietary supplements (U.S. FDA, 2019a) as well as in medical devices, such as wound dressings and gels (U.S. FDA, 2019b). Chit is known for its mucoadhesive properties and its ability to stimulate cells of the immune system, which supports the value of investigating Chit NPs as vaccine adjuvants (Dedloff et al., 2019). For this purpose, it has long been used by the group with various antigens, such as the hepatitis B surface antigen (HBsAg) (Borges et al., 2008; Lebre et al., 2016; Jesus et al., 2017, 2018; Soares et al., 2018a,b; Bento et al., 2019), the protective antigen (PA) from anthrax (Bento et al., 2015) or antigens from *Schistosoma mansoni* (Oliveira et al., 2012). Nevertheless, in the literature, Chit NPs have also been tested as drug delivery systems, without considering its immunomodulatory activity. An example of this situation is the numerous studies with the encapsulation of insulin into chitosan particles (Al Rubeaan et al., 2016). Furthermore, although there are several studies evaluating Chit NP toxicity *in vitro*, most of them do not assess the dysregulation of the immune system function (immunotoxicity). From the ones that do, the results are frequently contradictory. These contradictions and ambiguity may be due to differences in the used Chit polymers or *in vitro* methodology, namely the cellular model, NP concentration and incubation period. Moreover, it has been observed that most of the studies do not properly characterize, or at least do not report, both the polymer and the derived NPs, nor use or report adequate controls to screen NP interferences or monitor the presence of endotoxin contamination (Jesus et al., 2019). Notably, in the context of Safe-by-Design (SbD) of new polymeric NPs for drug delivery, it is necessary to rely on assertive results of immunotoxicity and hemocompatibility, obtained with properly characterized polymeric NPs.

The aim of this study is to explore the influence of the DDA of Chit polymer on immunotoxicity and hemocompatibility of Chit NPs. Therefore, murine RAW 264.7 cells, Peripheral Blood Mononuclear Cells (PBMCs) and whole blood were used as representative *in vitro* models for the immune system.

Nitric oxide (NO), reactive oxygen species (ROS) and cytokine production, cell viability, hemolysis, coagulation times and platelet aggregation were studied using appropriate controls under endotoxin-free conditions, and following protocols and recommendations, with slight changes, described by the European Nanomedicine Characterization Laboratory (EU-NCL) (EU-NCL, 2019).

## Materials and Methods

### Chitosan Polymers

Two different low molecular weight (LMW) Chitosans (ChitoClear™) were kindly donated by Primex BioChemicals AS (Avaldsnes, Norway). According to the supplier's specifications, one Chit had a lower deacetylation degree (DDA) and a viscosity of 13 cP (1% solutions in 1% acetic acid), while the other had higher DDA and a viscosity of 71 cP. Their exact DDA was found to be 80 and 93%, respectively, using the methodology described below.

The polymers were purified using a routine technique used in our laboratory and previously described by us (Lebre et al., 2019). Briefly, 1 g of Chit was suspended in 10 mL NaOH (1 M) solution. This suspension was heated between 40 and 50°C under continuous magnetic stirring for 3 h. After this time, the suspension was allowed to reach room temperature and was filtered using a Buchner funnel. Insoluble Chit on the filter was washed with water and then recovered to be further dissolved in 200 mL of 1% acetic acid solution and stirred for 1 h at room temperature. The Chit solution was then filtered through a 0.45 μm filter and 1 M NaOH solution was used to adjust the pH of the filtrate to pH 8.0 to precipitate Chit. The precipitate was then washed with water through three consecutives 30 min centrifugations at 4500 × g. The precipitate was recovered and freeze dried. To note that deionized water was used to obtain the purified polymer for the first experiments, optimization of the NP production method and physicochemical characterization, while LPS-free water was used to obtain LPS-free chitosan for cell *in vitro* studies. The purified polymers were used in all the methods described below.

Chit deacetylation degree and mean molecular weight were obtained by nuclear magnetic resonance (^1^H-NMR) and by size exclusion chromatography (SEC), respectively.

Deacetylation degree was determined as previously described (Lavertu et al., 2003). The DDA was calculated using the peaks of proton at the position 1 of deacetylated (H1D) and acetylated (H1A) monomer:

(1) $$\begin{array}{r} \text{DDA }\left(\%\right)=\left(\frac{\text{H}1\text{D}}{\text{H}1\text{D}+\text{H}1\text{A}}\right)\;\times 100 \end{array}$$

where H1D is shifted at 5.21 ppm and H1A at 4.92 ppm.

For Chit molecular weight (MW) analysis, two types of Chit polymers (before and after purification) were dissolved in 0.1 M acetic buffer (pH 4.0) containing 0.3 M NaCl to obtain 1 mg/mL solutions. Then they were filtered through 0.22 μm filters and collected in the chromatographic sample vials. For each analysis, 100 μL were injected at a flow rate of 1 mL/min at room temperature. Each sample was measured in triplicate. The interpretation of the obtained results was done using Mnova software.

Chit polymer particle size (micrometer range) was also characterized in acetate buffer and cell culture media using Beckman Coulter LS 13 320 Laser Diffraction Particle Size Analyzer (Beckman Coulter Inc., Brea, CA, USA).

### Preparation and Characterization of Chitosan Nanoparticles

To prepare both Chit NPs, each of the polymers (Chit 80% DDA and Chit 93% DDA) were dissolved at 0.1% (w/v) concentration in 1% (v/v) of acetic acid, and the pH was further adjusted to 4.6–4.8 using 10 N NaOH. Chit NPs spontaneously formed upon dropwise addition of 1.750 mL of sodium tripolyphosphate (TPP, 0.16% w/v) to 10 mL of Chit solution under high-speed homogenization. The final suspension remained in maturation during 30 min under magnetic stirring.

Chit NPs produced with Chit with 80% DDA were concentrated, washed with LPS-free water and concentrated again by centrifugation using Vivaspin 20 centrifugal concentrator (MWCO 300 kDa, 3,000 g). Chit NPs produced with Chit with 93% DDA were concentrated by centrifugation at 10,000 g (15 min) and centrifuged again at 7,000 g (15 min) with LPS-free water.

To evaluate if all the Chit polymer used for the NP production was effectively cross-linked with TPP, Cibacron Brilliant Red 3B-A dye assay (Muzzarelli, 1998) was used to quantify the free Chit that remained in solution after NPs preparation. The quantification was performed in 3 mL of the supernatants obtained by the previously described centrifugations, which were added to 100 μL of glycin/HCl buffer, 1 mL of the dye solution (0.015% dye in water, w/v) and 900 μL of ultra-pure water. The samples were left for 20 min in agitation and then the absorbance was read at 575 nm. The quantification was performed by interpolating the values with the values from a calibration curve ranging from 0.0004 to 0.0020% of Chit. The concentration of the Chit NPs was calculated subtracting to the initial mass of the chit used to prepare the NPs, the mas of free chitosan.

Delsa™ Nano C particle analyzer (Beckman Coulter, CA, USA) was used to measure NP size by dynamic light scattering (DLS) and the zeta potential through electrophoretic light scattering (ELS). Samples comprised the aqueous concentrated dispersions obtained after centrifugation, which were diluted with water before the measurements.

Concentrated samples of Chit NPs were tested for physicochemical stability when dispersed in cell culture media at 37°C for a maximum of 24 h. The resulting particle size and zeta potential were evaluated in Delsa™ Nano C particle analyzer (Beckman Coulter, CA, USA).

Images of Chit NPs, were acquired by two microscopy techniques. Transmission Electron Microscopy (TEM) using a FEI-Tecnai G2 Spirit Biotwin, (20–120) kV microscope (FEI company, Hillsboro, OR, USA) with NPs dispersed in water and subsequently dried out in the grid and observed with no contrast. For the second microscopy technique, a High resolution Scanning Electron CryoMicroscope (CryoSEM) (JEOL JSM 6301F/ Oxford INCA Energy 350/ Gatan Alto 2500) was used. The NP suspension was rapidly cooled in slush nitrogen, fractured and sublimated for 120 s at −90°C, before coating with Au/Pd. The sample was studied at −150°C.

For *in vitro* immunotoxicity studies, Chit purification and Chit NP production were conducted under endotoxin-free conditions following a methodology already published by our group (Lebre et al., 2019). All the reagents involved in NP production were tested with an endotoxin detection kit (Pyrochrome® Endpoint Chromogenic Endotoxin Testing, maximum sensitivity of 0.001 EU/mL, Associates of Cape Cod, Inc., East Falmouth, MA, USA) according to manufacturer's instructions.

### *In vitro* Studies With RAW 264.7 Cell Line

RAW 264.7 cell line (ATCC® TIB-71™) was acquired from ATCC (Manassas, VA, USA), cultured in Dulbecco's modified eagle's medium (DMEM, supplemented with 10% heat inactivated fetal bovine serum (FBS), 1% Penicillin/Streptomycin, 10 mM HEPES and 3.7 g/L sodium bicarbonate) and used until passage 18.

#### Cell Viability

The Cell viability of Chit NP and polymers was evaluated in RAW 264.7 cells using the 3-(4,5-dimethylthiazol-2-yl)-2,5-diphenyltetrazolium bromide (MTT) assay, performed in 96-well plates and cells plated at a density of 2 × 10^4^ cells per well. Serial dilutions of Chit NPs and Chit polymers ranged from 312 to 5,000 μg/mL final concentration in the well were incubated with the cells for 24 h, at 37°C and 5% CO_2_. Simultaneously, the NPs solvent (supernatant from the last washing centrifugation) and the polymer solvent (acetate buffer) were also tested in a dilution equivalent to the most concentrated samples. Then, 20 μL of MTT solution (5 mg/mL, in PBS) were added to each well and incubated for additional 1 h 30 min. To ensure the dissolution of the formazan crystals, cell culture medium was replaced by 200 μL of dimethyl sulfoxide (DMSO).

The resultant colored solution OD was measured at 540 nm and 630 nm. Cell viability (%) was calculated by the following equation:

(2) $$\begin{array}{r} \text{Cell viability }\left(\%\right)=\frac{\left(\text{OD sample }\left(540\text{ nm}\right)-\text{OD sample }\left(630\text{ nm}\right)\right)}{\left(\text{OD control }\left(540\text{ nm}\right)\;-\text{ OD control }\left(630\text{ nm}\right)\right)}\times 100\; \end{array}$$

The half maximal inhibitory concentration (IC50) of NPs that cause death or inhibition of the growth of 50% of cells was calculated by using the Log (NP concentration) vs. normalized response - variable slope analysis for the non-linear fit using Prism 6.0 (GraphPad Software, San Diego, CA, USA).

Interference controls were performed to guarantee the validity of the assay with the samples as suggested by Rösslein et al. (2015). Therefore, NPs and polymers in cell culture media without cells were plated in 96-well plates and the absorbance was measured (540 and 630 nm).

#### Production of Reactive Oxygen Species

The production reactive oxygen species (ROS) was assessed using the dichlorofluorescein diacetate (DCFH-DA) probe (Molecular Probes®, Life Technologies, Eugene, OR, USA). RAW 264.7 cells were incubated in black 96-well plates for 24 h at 37°C and 5% CO_2_, at a density of 0.5 × 10^5^ cells per well.

After that, serial dilutions of Chit NPs and Chit polymers (38 μg/mL to 156 μg/mL) were incubated with the cells in DMEM for 24 h at 37°C and 5% CO_2_, to evaluate ROS stimulation. The NPs solvent (supernatant from the last washing centrifugation) and the polymer solvent (acetate buffer) were also tested in a dilution equivalent to the most concentrated samples. Lipopolysaccharide (LPS, 1 μg/mL, from Salmonella enterica serotype minnesota, Sigma-Aldrich, Saint Louis, MO, USA) was used as a positive control or in combination with the same NP and polymer concentrations to test if the NPs were able to inhibit LPS stimulated ROS production.

Then, the cell culture medium was replaced by DCFH-DA (50 μM) in serum-free DMEM and the cells were incubated for additional 2 h at 37°C and 5% CO_2_. The resulting fluorescence was read at 485/20 (excitation) and 528/20 nm (emission) wavelengths.

To calculate the stimulation of ROS production (fluorescence fold increase) or the inhibition of ROS production (%) upon stimulation with LPS apply the following Equation (3) and (4), respectively.

(3) $$\begin{array}{r} \text{ROS production}=\frac{\text{Fluorescenc}{\text{e}}_{\text{SAMPLE}}}{\text{Fluorescenc}{\text{e}}_{\text{NEGATIVE CONTROL}}} \end{array}$$

(4) $$\begin{array}{r} \text{ROS inhibition}\left(\%\right)=\frac{\text{Fluorescence}{\;}_{\text{SAMPLE}}}{\text{Fluorescence}{\;}_{\text{POSITIVE CONTROL}}}\times 100\; \end{array}$$

Interference controls were performed to guarantee the validity of the assay with the samples. Therefore, NPs and polymers in cell culture media without cells were plated in black 96-well plates and all procedures were followed as in the original assay described.

#### Nitric Oxide Production

Nitric oxide (NO) has a short half-life in oxygen-containing aqueous solutions, often attributed to a rapid oxidation to nitrite. Therefore, NO production by RAW 264.7 cells was estimated based on nitrite quantification using the Griess reagent [1% (w/v) sulphanilamide mixed with 0.1% (w/v) naphthylethylenediamine dihydrochloride (1:1), both solutions previously dissolved in 2.5% (v/v) phosphoric acid].

RAW 264.7 cells were incubated in 48-well plates at a density of 2.25 × 10^5^ cells per well for 24 h at 37°C and 5% CO_2_. After that, cell culture medium was replaced by serial dilutions of Chit NPs and Chit polymers (38–156 μg/mL), diluted in cell culture medium without phenol red, and cells incubated for 24 h at 37°C and 5% CO_2_. The NPs solvent (supernatant from the last washing centrifugation) and the polymer solvent (acetate buffer) were also tested in a dilution equivalent to the most concentrated samples. LPS was used as a positive control (1 μg/mL). To test whether the NPs were able to inhibit LPS stimulated NO production, the same NP and polymer concentrations were incubated together with LPS (1 μg/mL).

After that, 100 μL of each cell supernatant were collected and plated in a 96-well plate and combined with an equal volume of the Griess Reagent. Several sodium nitrite solutions (0 μM to 80 μM) were also plated in duplicate to perform the calibration curve. The absorbance (Abs) of the samples was measured at 550 nm and the NO concentration (μM) was extrapolated from the calibration curve.

To calculate the inhibition of NO production upon stimulation with LPS, the Equation (5) was used.

(5) $$\begin{array}{r} \text{NO inhibition}\left(\text{\%}\right)\text{​}=\text{​}\frac{[\text{NO}]\;(\mu \text{M}){\;}_{\text{SAMPLE}}}{[\text{NO}]\;(\mu \text{M}){\;}_{\text{POSITIVE CONTROL}}}\times 100 \end{array}$$

Interference control was performed to guarantee the validity of the assay with the samples containing the particles. Therefore, 100 μL of NPs and polymers in DMEM without phenol red and without cells were plated in 96-well plates. Additionally, the NO calibration curve was performed in the presence of NPs and polymers, by plating in 96-well plates 50 μL of the samples and 50 μL of the standards used in calibration curve. Then, an equal volume of the Griess Reagent was added to each well and the absorbance was read as described above. This interference control was made at least in duplicate.

### *In vitro* Studies With Peripheral Blood Mononuclear Cells

#### PBMC Isolation

Peripheral blood (buffy coat) was kindly given by IPST, IP (Coimbra, PT) and was obtained from healthy donors in heparinized syringes followed by serum depletion. PMBCs were isolated on a density gradient with Lymphoprep (Axis-Shield, Dundee, SCT) according to the provider's guidance protocol, with minor modifications. Briefly, the blood dilution performed was 1:5 (v/v) in 0.9% sodium chloride, the centrifugation step was performed at 1,190 × g for 20 min (20°C) and the mononuclear cell dense ring was collected and washed with PBS (pH = 7.4 at 37°C) through consecutive centrifugations (487 × g, 10 min, 20°C) until the supernatant was clear. At the end, cells were suspended in Roswell Park Memorial Institute Medium (RPMI 1640) supplemented with 1% Penicillin/Streptomycin and 10% heat-inactivated FBS.

#### Cell Viability

Chit NP and polymer toxicity in PBMCs was assessed by MTT as described previously for RAW 264.7 cells, with some modifications. Briefly, cells were plated at a concentration of 7.5 × 10^6^ cells/well, test samples ranged from 2.44 to 5,000 μg/mL and MTT incubation was prolonged for 4 h. To ensure the dissolution of the formazan crystals, cell culture plates were centrifuged (800 × g, 25 min, 20°C) and 180 μL/well of the culture medium were replaced by an equal volume of DMSO.

Cell viability results obtained with the MTT assay were confirmed with propidium iodide (PI) assay, using four different NP concentrations. Cells incubated with the NPs were centrifuged (800 × g, 25 min, 20°C), resuspended in PBS and collected for flow cytometry analysis (BD FACSCalibur, BD Biosciences, Bedford, MA, USA). A volume of 2 μL of PI solution was added immediately before the analysis to achieve a final concentration of 0.5 μg/mL.

#### Cytokine Secretion

To analyze the cytokine secretion induced by Chit NPs, cells were plated in 96-well plates at a density of 2.5 × 10^5^ cells per well. Chit NPs and polymers (100 μg/mL) and positive controls (LPS 2 ng/mL, Con A 5 μg/mL) were incubated with the cells for 24 h, at 37°C and 5% CO_2_. Then, cell culture plates were centrifuged (800 × g, 25 min, 20°C) and the supernatants were collected for Enzyme-Linked Immunosorbent Assay (ELISA) according to manufacturer's instructions (Human TNF-α and IL-6 Standard TMB ELISA Development Kit, Peprotech, NJ, USA).

Interference controls were performed to guarantee the validity of the assay with the samples. Therefore, NPs and polymers in RPMI were incubated for 24 h, at 37°C and 5% CO_2_ without cells, in the presence of several concentrations of cytokine standards (TNF-α and IL-6), as used in the ELISA calibration curve. The same concentrations of each cytokine were also incubated in RPMI in the absence of the samples. After that time, supernatants were collected and analyzed by ELISA as described for the samples with cells, according to manufacturer's instructions.

### *In vitro* Studies With Human Blood

Blood was collected from healthy volunteers at the Clinical Laboratory Analysis of Faculty of Pharmacy (University of Coimbra, Portugal). A written informed consent was obtained from all participants. Anonymous blood samples were used by the researchers for the hematological *in vitro* assays.

#### Hemolysis Assay

To perform hemolysis assay, plasma free hemoglobin (PFH) concentration was required to be below 1.0 mg/mL. Whole blood was collected in heparinized tubes and diluted in PBS to adjust total blood hemoglobin (TBH) concentration to 10 mg/mL ± 2 mg/mL (TBHd). A volume of 100 μL of cyanmethemoglobin (CMH, blank), Chit NP suspensions, Chit polymer suspensions, PBS (negative control), Triton-X-100 (positive control) or NPs solvent (interference control) were added to 700 μL of PBS in different tubes. Then, 100 μL of TBHd was added and incubated at 37°C for 3 h ± 15 min. NPs were also incubated with PBS without blood to evaluate the possible NP interference with the assay. Then, the mixture was centrifuged at 800 × g for 15 min. A volume of 100 μL of supernatant and 100 μL of CMH reagent were added to a 96-well plate. The CMH reagent was prepared by mixing 1,000 mL Drabkin's reagent and 0.5 mL of 30% Brij 35 solution (Sigma-Aldrich, Saint Louis, MO, USA). The absorbance (OD) was read at 540 nm. The percentage of hemolysis was calculated using the following equation:

(6) $$\begin{array}{r} \text{Hemolysis }\left(\%\right)=\frac{\left(\text{OD sample }\left(540\text{ nm}\right)-\text{OD negative control }\left(540\text{ nm}\right)\right)}{\left(\text{OD TBHd }\left(540\text{ nm}\right)-\text{OD negative control }\left(540\text{ nm}\right)\right)}\times \;100\; \end{array}$$

#### Coagulation Assay

The two pathways of blood coagulation, the activated partial thromboplastin time (APTT) and the prothrombin time (PT) were separately tested. Blood was collected using sodium citrate tubes and the plasma was obtained by centrifugation of the blood at 2500 × g for 10 min. Plasma (450 μL) was incubated with a volume of 50 μL of Chit NPs and Chit polymer suspensions (two final concentrations: 0.1 and 1 mg/mL), for 30 min at 37°C. Then, samples were evaluated using Bio-TP LI (PT) and Bio-CK (APTT) kits (Biolabo S.A.S., Maizy, France) according to manufacturer's instructions, in an Option 4 plus coagulation analyzer (BioMérieux, Marcy-l'Étoile, France).

#### Platelet Aggregation Assay

Platelet-rich plasma (PRP) was obtained from blood collected in sodium citrate tubes, and centrifuged at 200 × g for 16 min. Platelet-free plasma (PFP) was obtained after blood centrifugation at 2,500 × g for 10 min, followed by plasma centrifugation at 18,000 × g for 5 min. A volume of 100 μL of PRP or 100 μL of PFP were added to 96-well plates and incubated for 5 min at 37°C. A volume of 25 μL of Chit NPs at 2 mg/mL, saline solution (negative control) and calcium chloride 0.25 M or collagen (positive controls) were added to the wells with PRP and incubated for 30 min at 37°C. Then, 4 μL of Giemsa dye was added to each well and incubated for 5 min. Finally, a 1:200 dilution with saline solution was applied for platelet counting (PC) using a light microscope. Chit NPs were also incubated with PFP to evaluate the NPs interference in plasma.

The percentage of platelet aggregation was calculated using the equation 7.

(7) $$\begin{array}{r} \text{Platelet aggregation }\left(\%\right)=\frac{\left(\text{PC negative control }-\text{ PC sample}\right)}{\text{PC negative control}}\;\times \;100 \end{array}$$

### Statistical Analysis

Results were expressed as mean ± standard error of the mean (SEM). Prism 6.0 (GraphPad Software, San Diego, CA) was used for all statistical analysis. Statistical significance was assessed using one-way ANOVA.

## Results

### Physicochemical Characterization of Polymers and Nanoparticles

#### Polymer Purification Reduces the Molecular Weight of the Lower Deacetylation Degree Chitosan

The characterization of the polymers used and the nanoparticulate delivery system developed is critical to prevent erroneous interpretations of resultant immunotoxicity findings. Different Chit characteristics can have different biological effects. Unfortunately, most studies addressing biological activity of Chit NPs lack the used polymer characterization, which also restricts comparisons among studies.

The two Chit polymers used in this study were purified under endotoxin-free conditions to eliminate possible contaminants. Since the purification process involves harsh conditions, namely heating the polymer suspension in NaOH 1 M, their DDA and MW were assessed before and after purification and the results presented in Table 1A. Chit deacetylation experienced no significant alterations, resulting in polymers with 80 and 93% DDA (Chit 80% and Chit 93%, respectively). In contrast, the MW before and after purification for the lower DDA Chit (Chit 80%) was altered. An important decrease from 168 to 49 kDa is compatible with the fact that lower DDA Chit has higher enzymatic and acid hydrolysis degradation rate (Kurita et al., 2000; Vårum, 2001; Szymanska and Winnicka, 2015).

**Table 1:** Physicochemical characterization of Chit polymers and NPs. (A) Polymer molecular weight (MW), deacetylation degree (DDA), and size in acetate buffer and after resuspension in DMEM and RPMI at 37°C (Mean ± SEM). (B) Chit 80% and Chit 93% NPs size, polydispersity index and zeta potential (ζ), in water and after resuspension DMEM and RPMI at 37°C *(Mean* ± *SEM)*. (C) Endotoxin contamination evaluated with the Pyrochrome^®^ kit for Chit 80% NPs, Chit 93% NPs, Chit 80%, and Chit 93% and TPP solution. Endotoxin contamination of pyrogen-free water was also evaluated for comparison. Mean ± SEM; n = 3 (three different batches).

**A**									
	**MW (kDa) (*****n*** **=** **1–3)**		**DDA (%) (*****n*** **=** **1)**		**Size (μm) (*****n*** **≥** **3)**				
					**Acetate buffer**	**DMEM**		**RPMI**	
	**Non-purified**	**Purified**	**Non-purified**	**Purified**		**1 h**	**24 h**	**1 h**	**24 h**
**Chit 80%**	168	49	78	80	612 ± 40	529 ± 39	541 ± 49	628 ± 94	479 ± 58
**Chit 93%**	127	122	94	93	608 ± 23	590± 37	518 ± 57	555 ± 50	525 ± 53

**Table:** 

**B**									
		**Water**	**DMEM**		**RPMI**	
			**1 h**	**24 h**	**1 h**	**24 h**
**Chit 80% NPs**	**Size (nm)**	127 ± 5	109 ± 29	133 ± 22	116 ± 29	368 ± 141
**(*****n*** **≥** **3)**	**PDI**	0.28 ± 0.01	0.72 ± 0.03	0.62 ± 0.11	0.47 ± 0.08	0.52 ± 0.03
	**ζ** **(mV)**	+29.0 ± 1.0	−4.9 ± 0.2		−2.1 ± 0.4	
**Chit 93% NPs**	**Size (nm)**	292 ± 52	106 ± 20	147 ± 74	321 ± 48	327 ± 131
**(*****n*** **≥** **3)**	**PDI**	0.18 ± 0.03	0.49 ± 0.14	0.48 ± 0.21	0.91 ± 0.13	0.76 ± 0.16
	**ζ** **(mV)**	+20.0 ± 6.0	−3.9 ± 0.6		−4.4 ± 0.5	

**Table:** 

**C**									
	**Endotoxin (EU/mL) (*****n*** **=** **3)**			**Endotoxin (EU/mL) (*****n*** **=** **3)**	
	**Mean**	**SEM**		**Mean**	**SEM**
**Pyrogen-free water**	0.05861	0.00607	**TPP solution**	0.05492	0.00814
**Chit 80%**	0.06224	0.01815	**Chit 80% NPs**	0.07166	0.01246
**Chit 93%**	0.06820	0.03252	**Chit 93% NPs**	0.08844	0.03189

Chit is soluble in acidic conditions, which is incompatible with cell culture as it leads to cell death. Therefore, *in vitro* studies with Chit polymers (purified raw material) were performed with Chit suspended in acetate buffer (pH = 5.0), further diluted in cell culture medium (156.25 μg/mL). Particle size in acetate buffer and cell culture media is illustrated in Table 1A. The mean average size of these particle suspensions was around 500 μm in all situations.

#### Chitosan With Higher Molecular Weight and Deacetylation Degree Leads to Larger-Sized NPs

Chit NPs were successfully produced by ionic gelation method, using TPP as the crosslink (Chit 80% NPs and Chit 93% NPs). These NPs were isolated and concentrated in water. Importantly, the analysis of the first supernatants revealed that more than 99% of the Chit used in the production was retained in the NPs. This result was important to calculate Chit NP concentration.

**Figure 1:**
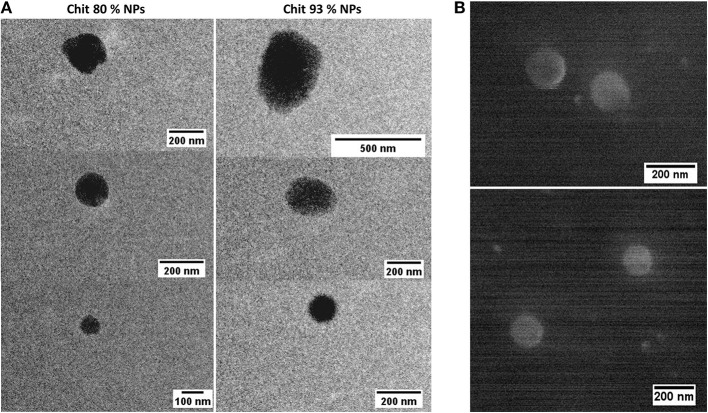
Chit NP illustration by Electron Microscopy. **(A)** Transmission Electron Microscopy (TEM) of Chit 80% NPs, presented in the left side column and of Chit 93% NPs, presented in the right side column. **(B)** Scanning Electron Microscopy (SEM) of Chit 80% NPs.

After isolation and concentration, NP mean particle size, polydispersity index (PDI), and zeta potential (ζ) were determined by DLS and ELS, respectively, and are summarized in Table 1B. Results illustrate the effect of the different Chit on the NP characteristics. In fact, the same methodology, when applied to Chit polymers with different DDA and MW, resulted in NPs with different sizes. Lowering the DDA from 93 to 80% caused the mean particle size to fall from 292 to 127 nm. These average particle sizes were illustrated by TEM and SEM analysis. The round shape of the NPs was the second conclusion inferred by observing the images (Figures 1A,B) of both techniques. Concerning zeta potential, both Chit 93% and Chit 80% NPs presented a positive charge when dispersed in deionized or pyrogen-free water, although slightly more positive for Chit 80% NPs (+20 and +29 mV, respectively).

**Figure 2:**
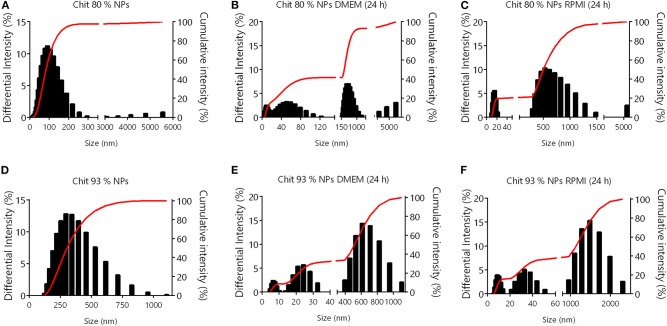
NP size distribution by DLS. **(A)** Chit 80% NPs in water; **(B)** Chit 80% NPs after 24 h in DMEM medium; **(C)** Chit 80% NPs after 24 h in RPMI medium; **(D)** Chit 93% NPs in water; **(E)** Chit 93% NPs after 24 h in DMEM medium; **(F)** Chit 93% NPs after 24 h in RPMI medium.

Due to the complexity of cell culture media, and the variability of their supplementation, results from NP colloidal system characterization in water are not transposable to *in vitro* conditions (Moore et al., 2015). Chit NPs were therefore characterized in cell culture media to understand the changes that NPs experience during *in vitro* studies. Chit 80% and Chit 93% NPs were added to DMEM and RPMI (containing FBS) at 37°C at a concentration of 156.25 μg/mL for further size and PDI measurement after 1 and 24 h, and zeta potential measurement after 1 h (Table 1B). Even though the DLS methodology for size analysis in complex media (such as cell culture medium) has limitations, it can give us some insights about changes occurring to the different Chit NPs. Most notably, the suspension of both Chit NPs in RPMI and DMEM resulted in increased PDI, meaning an increase of the size heterogeneity. The zeta potential of the NPs decreased when measured in both cell culture media (ranging from −2 to −5 mV) (Table 1B). This change induced by the adsorption of negatively charged proteins from the medium, to positively charged Chit residues, forms a protein corona, decreasing the suspension stability. Under these conditions, the appearance of aggregates is inevitable which is part of the explanation for the PDI increment. To further complement the information given by the PDI and intensity average size, graphics from size distribution illustrate the different size populations of Chit 80% NPs and Chit 93% NPs in the different media (Figure 2). In the water, the size of both NP was distributed over a single peak (Figures 2A,D), while in cell culture media, there were at least three independent peaks (Figures 2B,C,E,F). We can hypothesize that the alterations observed in cell culture media size dispersion, including smaller and bigger size populations simultaneously, were induced not only by the presence of proteins, but also by the high ion content in comparison to water (Moore et al., 2015). Furthermore, as the media composition is different between RPMI and DMEM, the observed changes in the NP size distribution were not similar. A comparable phenomenon was described by Yang et al. (2018) for silica and silica coated nanoparticles, whose great stability in buffered saline was not kept in cell culture media, where the authors verified the erosion of surface silica by DMEM ingredients.

#### Endotoxin-Free Conditions Guarantee the Production of LPS-Free Nanoparticles

The last step of characterization was related to endotoxin contamination. As previously mentioned, Chit polymers were purified by a method published by our group (Lebre et al., 2019). The method allows the obtainment of endotoxin-free chitosan, proved by two methods: Limulus Amebocyte Lysate (LAL) test and the absence of IL-6, secreted by dendritic cells (DCs), cultured in the presence of chitosan. The chitosan does not induce IL-6 secretion by DCs and endotoxins do that stimulation. Furthermore, for *in vitro* immunotoxicity studies, the NP production was performed under those conditions, to avoid endotoxin contamination, as the presence of these molecules can easily lead to false positive results. To assure that Chit purification and Chit NP production were successfully achieved, both Chit polymers and NPs as well as the pyrogen-free water and the TPP solution used for NP production, were submitted to LAL test. Importantly, before establishing the methodology for endotoxin quantification with Pyrochrome® testing kit, all recommended tests to evaluate sample interference with LAL test were done to guarantee the suitability of the LAL test for Chit NPs, as described in the manufacturer's instructions. The results were presented in Table 1C, and show that all tested samples were not significantly different from pyrogen-free water, the negative control, and all were far below 0.25 EU/mL, which is the limit for water for injection according to main health authorities (Ph. Eur. 9.0, 2019). Thus, it was demonstrated that the process and conditions used to minimize the contamination and remove existent endotoxins during Chit purification and NP production was effective, and that Chit polymers and NPs used in immunotoxicity tests were indeed LPS-free, supporting the reliability of the results.

### *In vitro* Studies With RAW 264.7 Cell Line

The monocyte/macrophage-like RAW 264.7 cells have been widely used for 40 years, as a suitable *in vitro* model, since they present unique phenotype and functional characteristics of macrophages (Roberts et al., 2018). Nevertheless, these cells should be used carefully since their functional stability is not maintained at high passage number. Indeed, a recent article mentions the phenotype and functional characteristics to remain stable from passage 10 to 30 (Roberts et al., 2018), and the American Type Culture Collection (ATTC) recommends to use them until passage 18.

#### Chitosan Nanoparticles Are More Cytotoxic for RAW 264.7 Cells Than Chitosan Polymers

**Figure 3:**
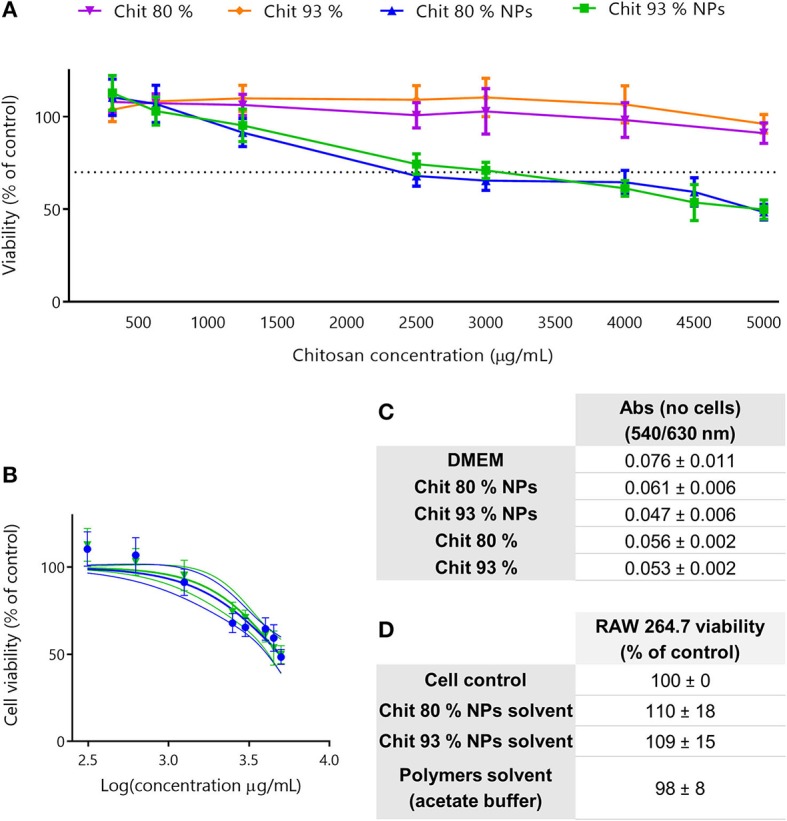
Cell viability studies in RAW 264.7 cell line. **(A)** Cell viability decrease induced by different concentration of Chit 80% NPs, Chit 93% NPs, Chit 80%, and Chit 93% polymers evaluated by MTT assay after 24 h incubation. Dotted line represents the 70% of cell viability. **(B)** Nonlinear regression analysis of the cell viability data, allowing the extrapolation of IC50 values (4,949 μg/mL for Chit 80% NPs and 4,858 μg/mL for Chit 93% NPs). No statistical difference was found between Chit 80% NPs IC50 and Chit 93% NPs IC50 calculated using extra sum-of-squares F test. **(C)** Evaluation of possible NP and polymer interference with the wavelengths used to read MTT assay (540/630 nm) (Mean ± SEM, *n* = 3). **(D)** Evaluation of the cell viability resultant from the incubation of RAW 264.7 with the NPs solvent and polymers solvent (% of control). Results are expressed as mean ± SEM, obtained from a minimum of three independent experiments, each performed in triplicate (*n* ≥ 3).

The evaluation of the cytotoxic profile of Chit NPs and polymers was performed using the MTT metabolic activity assay, over a wide range of concentrations as illustrated in Figure 3. Results showed that Chit 80% and Chit 93% polymers were not cytotoxic in the concentration range tested (purple and orange lines, respectively), while Chit 80% and Chit 93% NPs induced significant decrease of cell viability above 2,500 and 3,000 μg/mL, respectively (Figure 3A). Based on the nonlinear regression analysis of the cell viability data of the Chit NPs, non-significant differences were found for the IC50 of Chit 80% NPs and Chit 93% NPs (Figure 3B).

The reduction of the reagent MTT by cells leads to the generation of insoluble crystals of formazan that once dissolved in DMSO generate a purple signal (van Meerloo et al., 2011). Since it is a colorimetric assay, and although the cell medium with the testing sample was aspirated before solubilizing the formazan crystals, NP interferences with the readout were tested to validate the assay (Figure 3C). As it is possible to observe, the measured absorbance (Abs) was not increased by the presence of the NPs or polymer suspension. Additionally, to guarantee that the cell viability results were only related with the NP and polymers, and not with the solvents, the supernatants collected from the NPs last washing step with water, as well as the acetate buffer used to disperse the polymers, were also tested using MTT assay (Figure 3D). Results showed that the solvents did not cause any decrease in cell viability.

#### Both Chitosan Polymers Hamper Nitric Oxide Release After LPS Stimulation and Only the Lower Deacetylation Degree Chitosan Induces Oxidative Stress

Reactive oxygen species (ROS) are unstable molecules that easily react with other molecules and may cause damage to DNA, RNA, proteins and ultimately lead to cell death, when accumulated (Schieber and Chandel, 2014).

**Figure 4:**
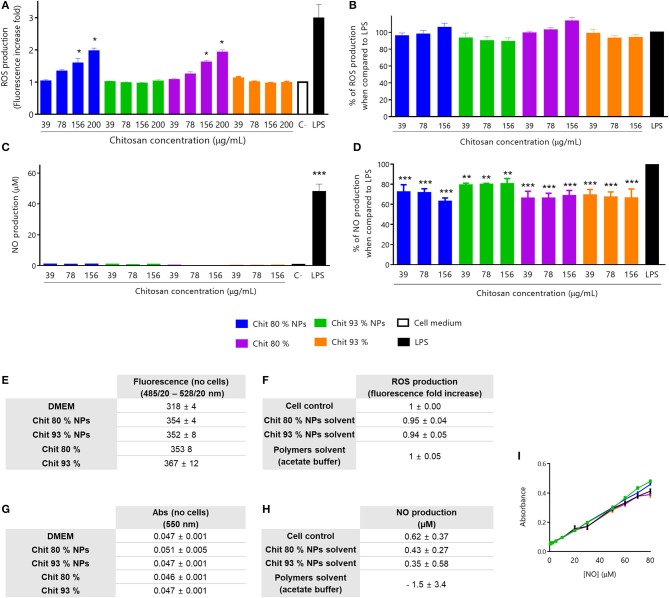
Immunotoxicity assays in RAW 264.7 cell line. All assays were performed with non-cytotoxic concentrations of NPs, polymers and controls (evaluated by MTT assay after every experiment). **(A)** ROS production stimulated by Chit 80% NPs, Chit 93% NPs and the respective polymers prepared in endotoxin-free and sterile conditions. For the experiment, test samples were incubated with RAW 264.7 cell line for 24 h, as well as LPS, as a positive control. Mean ± SEM; obtained from four independent experiments, each performed in triplicate (*n* = 4), *p < 0.05 compared to control. **(B)** Inhibition of ROS production by Chit 80% NPs, Chit 93% NPs and the respective Chit polymers prepared in endotoxin-free and sterile conditions. For the experiment, LPS and test samples were co-incubated with RAW 264.7 cell line for 24 h. Mean ± SEM, obtained from a minimum of seven independent experiments, each performed in triplicate (*n* ≥ 7). **(C)** NO production stimulated by Chit 80% NPs, Chit 93% NPs and the respective polymers prepared in endotoxin-free and sterile conditions. For the experiment, test samples were incubated with RAW 264.7 cell line for 24 h, as well as LPS, as a positive control. Mean ± SEM, obtained from a minimum of three independent experiments, each performed in triplicate (*n* ≥ 3), ***p < 0.001 compared to control. **(D)** Inhibition of NO production by Chit 80% NPs, Chit 93% NPs and the respective polymers prepared in endotoxin-free and sterile conditions. For the experiment, LPS and test samples were co-incubated with RAW 264.7 cell line for 24 h. Negative control (C–) was not co-incubated with LPS. Mean ± SEM, obtained from a minimum of three independent experiments, each performed in triplicate (*n* ≥ 3), **p < 0.01 and ***p < 0.001 compared to LPS control. **(E)** Evaluation of possible NP and polymer interference with the wavelength used to read ROS assay (Ex485/20 – Em528/20) (Mean ± SEM, *n* = 3). **(F)** Evaluation of the ROS production (fluorescence fold increase) induced by the NPs solvent and polymers solvent (*n* = 3). Data are presented as mean ± SEM. **(G)** Evaluation of possible NP and polymer interference with the wavelength used to read NO assay (550 nm) (Mean ± SEM, *n* = 3). **(H)** Evaluation of the NO production (%) induced by the NPs solvent and polymers solvent (% of control) (*n* = 3). Data are presented as mean ± SEM. **(I)** Control of interferences of **(A)** Chit NPs and **(B)** Chit polymers with known concentrations of NO without cells (Mean ± SEM, *n* = 3).

To evaluate the effect of Chit polymers and Chit NPs on ROS production by RAW 264.7 cells, four different concentrations were used. As it is possible to see in Figure 4A, only Chit 80% NPs and the respective Chit polymer were able to induce ROS production, under non-cytotoxic concentrations. The increase in ROS production was concentration dependent, however, for the concentration range tested, the effect was not as high as LPS-induced ROS production. On the other hand, Chit 93% NPs and polymer had no effect on ROS production by RAW 264.7 cells. Importantly, all tested conditions did not induce cellular death as confirmed by the MTT assay performed at the end of each experiment (Figure S1A). In order to have a more complete picture, studies were conducted to evaluate that the polymers and NPs would not play an inhibitory role in the production of ROS by cells stimulated with LPS. Therefore, increasing concentrations of Chit polymer or Chit NPs were incubated together with cells and 1 μg/ml of LPS. Results in Figure 4B show that no inhibitory effect was observed for any of the tested samples. Consequently, it was possible to conclude that Chit 80% NPs, Chit 93% NPs and the respective polymers, when used in non-cytotoxic concentrations (cell viability results are on Supplementary Figure S1B), were not able to reduce the LPS-induced ROS production.

The possibility of having the nanoparticles interfering with the methods should not be ruled out, leading to false positives or false negatives. So, to evaluate the interference of Chit NPs and Chit polymers in the fluorescence readouts, the ROS production assay was performed without cells and at the highest polymer and NPs concentrations. The values obtained for test samples were similar to the medium alone (Figure 4E), meaning that they do not interfere with ROS measurement. Additionally, the possible interference of solvents was also assessed under the same testing conditions and as shown in Figure 4F, no stimulation of ROS production, as the fluorescence increase fold values were around 1.

NO is an important inflammatory mediator released by macrophages during inflammation, being one of the main cytostatic, cytotoxic, and pro-apoptotic mechanisms of the immune response (Bosca et al., 2005). NO production by RAW 264.7 cell line was measured using the Griess reaction method. Again, all test samples were sterile and endotoxin-free in order to prevent false positive results, and used in adequate concentrations that did not affect cell viability (Cell viability study in Supplementary Figures 1C,D).

With the aim to evaluate whether one of the polymers or Chit NPs would be able to induce cells to produce NO, samples were incubated with the RAW 264.7 cells for 24 h and the results were presented in Figure 4C. None of the Chit NPs or polymer concentrations tested induced NO production. Additionally, to evaluate whether the NPs and polymers had an inhibitory effect on NO production when cells were stimulated by LPS, increasing concentrations of the polymers and NPs were incubated with cells and with 1 μg/ml LPS. The results shown in Figure 4D indicate that there was a slight but significant inhibitory effect on LPS-induced NO production, at all concentrations tested when compared to the LPS control. Since the Chit and Chit NP concentrations tested did not induce significant reduction in cell viability (Supplementary Figure 1D) we can exclude the hypothesis that it was a consequence of cellular death.

For all NPs, the possible interference with optical detection methods is a hypothesis that should be tested before doing the test itself. So, similar to ROS assay, the NO assay was performed in the presence of the test samples, without cells and the results were presented on Figure 4G. The solvent of the Chit NPs suspension or the chitosan polymer suspension were evaluated to understand if they also had an effect on NO production (Figure 4H). No interferences were observed in the readout, and the solvents were not able to induce NO production. An additional control was performed for NO production assay, to evaluate whether Chit and Chit NPs, due to their cationic charge, could be adsorbing NO at their surface, reducing the amount of NO quantified. Such phenomenon would provide an explanation for the NO production inhibition observed. To evaluate this hypothesis, we performed the NO calibration curve in the presence and absence of Chit NPs and polymers (Figure 4I). As shown, the NO curves are all overlapping, meaning no interferences from Chit NPs and Chit polymers were observed.

### *In vitro* Studies With Human Peripheral Blood Mononuclear Cells

PBMCs are a good model to study immune responses, since they secrete regulatory and pro-inflammatory cytokines and chemokines in the human body. *In vitro* cell viability experiments give an indication of a particle cytotoxic profile that may be observed *in vivo*.

#### Lower Deacetylation Degree Chitosan NPs Are More Cytotoxic for PBMCs

**Figure 5:**
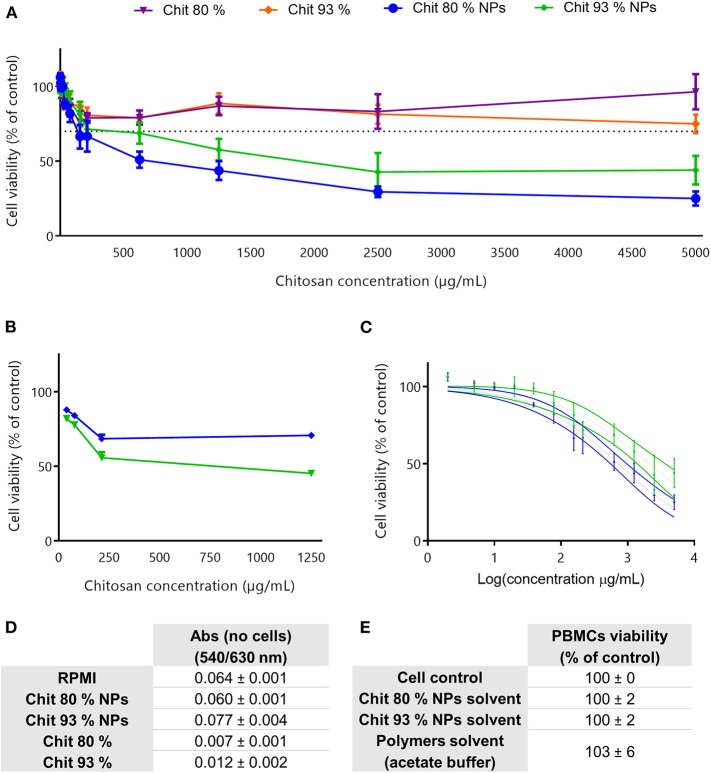
Cell viability studies in PBMCs and assay interference evaluation. **(A)** Cell viability decrease induced by different concentrations of Chit 80% NPs, Chit 93% NPs, Chit 80%, and Chit 93% in human PBMCs, evaluated by MTT assay following 24 h of incubation. Dotted line represents the 70% of cell viability. Results are expressed as mean ± SEM, obtained from four independent experiments, each performed in triplicate (*n* = 4). **(B)** Confirmation of MTT results by testing four different concentrations of Chit 80% NPs and Chit 93% NPs by flow cytometry using PI. Results are expressed as mean ± SEM, obtained from 1 to 4 independent experiments, each performed in duplicate (*n* = 1–4). **(C)** Nonlinear regression analysis of the cell viability data, allowing the extrapolation of IC50 values (720 μg/mL for Chit 80% NPs and 2104 μg/mL for Chit 93% NPs). Significant statistical difference between Chit 80% NPs IC50 and Chit 93% NPs IC50 calculated using extra sum-of-squares *F*-test. **(D)** Evaluation of possible NP and polymer interference with the wavelength used to read MTT assay (540/630 nm) (Mean ± SEM, *n* = 3). **(E)** Evaluation of the cell viability resultant from the incubation of PBMCs with the NPs solvent and polymers solvent (% of control) (Mean ± SEM, *n* = 4).

Similar to RAW 264.7 cell line experiments, Chit NPs and polymers were incubated with cells, in this case human PBMCs, and the cell viability was evaluated using the MTT assay. The results depicted in Figure 5A showed that Chit NPs were more cytotoxic than the respective polymers.

Comparing the results achieved between the two NPs, Chit 80% NPs showed a tendency to be more cytotoxic than the Chit 93% NPs. This difference was further confirmed with the PI assay, where the cell membrane integrity rather than the metabolic activity was evaluated (Figure 5B). A nonlinear regression of the MTT assay results clearly showed that Chit 80% NPs induced a more accentuated decrease in cell viability, with the 50% inhibitory concentration (IC50) calculated at ~720 μg/mL (Figure 5C). Chit 93% NPs showed a statistically different IC50, calculated to be 2,104 μg/mL.

To note, Chit NP and polymer highest concentrations tested during cell viability assessment in both RAW 264.7 and PBMCs were very high and do not correlate with concentrations required for *in vivo* assays. Nevertheless, 5,000 μg/mL is recommended in OECD guidelines for genotoxicity testing of chemicals (test guideline 487) as the maximum concentration to be tested when no cytotoxicity or precipitates are observed. In our case, these concentrations were needed to correctly calculate the IC 50. For the Chit polymers, even though the highest sample concentration was very thick, it did not induce toxicity below 70%, confirming the great biocompatibility of the Chit polymers.

As explained for the RAW 264.7 cell line, experimental controls were performed and the results are presented in Figures 5D,E. The absorbance readout showed no interference for formulations (equal Abs values) and the resultant cell viability following solvent incubation with PBMCs during 24 h showed comparable cell viability to the control.

#### LPS-Free Chitosan Nanoparticles Do Not Stimulate IL-6 and TNF-α Release by PBMC's

Cytokines participate in many physiological processes, mostly in the regulation of immune and inflammatory responses (Ai et al., 2013). Interleukin-6 (IL-6) is a pleiotropic cytokine (inflammatory and anti-inflammatory properties) able to modulate the activity of immune cells (Wang et al., 2017). Tumor necrosis factor-α (TNF-α) is a pro-inflammatory cytokine released from macrophages or activated T cells which plays a crucial role in many immune and inflammatory processes, such as proliferation, apoptosis, and cell survival (Cai et al., 2017).

**Figure 6:**
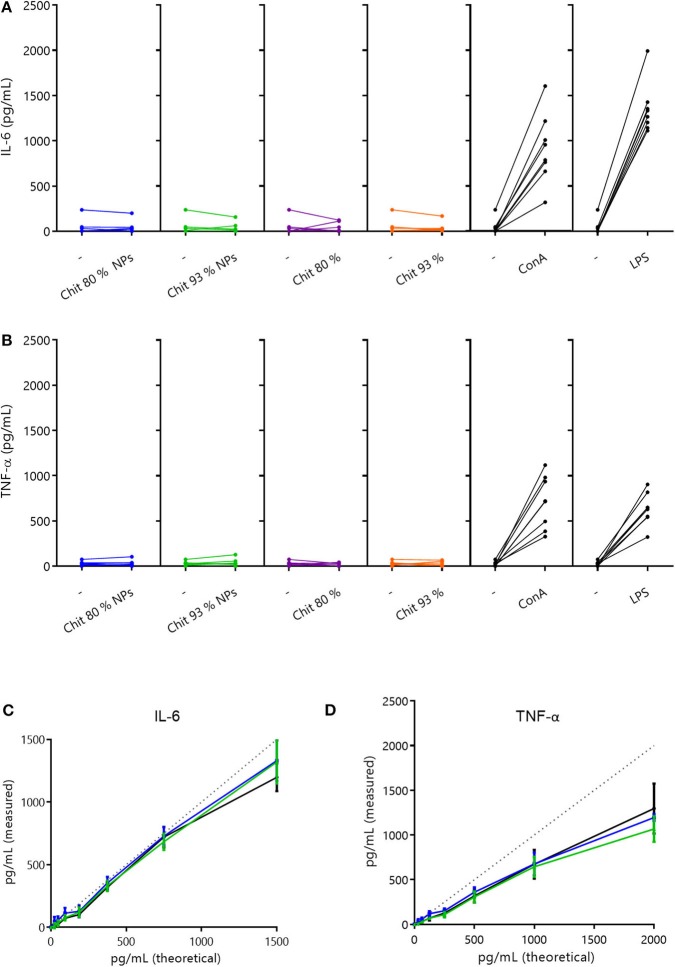
Cytokine secretion in PBMCs and interference evaluation. **(A,B)** Cytokine secretion induced by 100 μg/mL of Chit 80% and Chit 93% polymers and NPs on human PBMCs, after 24 h incubation (A- IL-6 and B- TNF-α). Cytokine quantification was performed by ELISA. Results illustrate the increase in cytokine production (Chit 80% NPs, Chit 93% NPs, Chit 80%, Chit 93%, ConA, LPS), when compared to the basal level (–). The experiment was repeated with blood from eight different donors (*n* = 8). **(C,D)** Evaluation of the Chit 80% and Chit 93% NPs ability to interfere with cytokine quantification when compared to the cell culture media (experiment without cells). The results of the cytokine quantification for the calibration curve in the presence of Chit 80% NPs and Chit 93% NPs were compared to the cytokine quantification of the calibration curve in simple cell culture media (C- IL-6 and D- TNF-α). Dotted lines represent the original calibration curve in ELISA diluent. Data are represented as mean ± SEM (*n* = 3).

In order to understand if Chit NPs and polymers were able to stimulate the release of these cytokines by human PBMCs, the cells were incubated with 100 μg/mL Chit test samples for 24 h and the secreted cytokine results, measured by ELISA, were depicted in Figures 6A,B. Results showed that neither Chit NPs nor Chit polymers stimulated the production of IL-6 and TNF-α, as no differences were found before and after incubation with test samples. Importantly, the use of positive controls such as LPS and Con A, give us an indication of the cell function regarding the cytokine we are analyzing. Notably, both positive controls significantly increased the cytokine secretion in PBMCs.

Additionally, since chitosan's positive charge favors cytokine adsorption, the possible interference of Chit NPs in cytokine quantification was tested. For that, Chit NPs suspended in cell culture medium were incubated with known concentrations of each cytokine (calibration curve) for 24 h, then centrifuged and supernatant cytokine content similarly quantified by ELISA. The percentage of cytokine quantification in cell culture medium incubated with the nanoparticles in comparison to cell culture medium without nanoparticles, can reveal if the cytokines adsorbed to the NPs, preventing their quantification. Interestingly, Figures 6C,D suggest that Chit NPs did not adsorb IL-6 nor TNF-α, since cytokine quantification was equal or above 100%. Thus, we can assume that the absence of TNF-α and for IL-6 production upon stimulation with Chit NPs and polymers was indeed due to the lack of the samples' ability to stimulate the cells, which strengthens the conclusion that they do not induce a pro-inflammatory cytokine response, at least when produced under endotoxin-free conditions.

### Hemocompatibility Assays

#### Chitosan Nanoparticles and Polymers Do Not Induce Hemolysis Even at High Concentrations

Hemolysis is characterized by the rupture of red blood cells (RBCs) and the release of their contents, ultimately leading to anemia, jaundice and renal failure (Dobrovolskaia et al., 2008). All materials entering the blood get in contact with RBCs and so the evaluation of the hemolytic ability of the biomaterials is of utmost importance.

**Figure 7:**
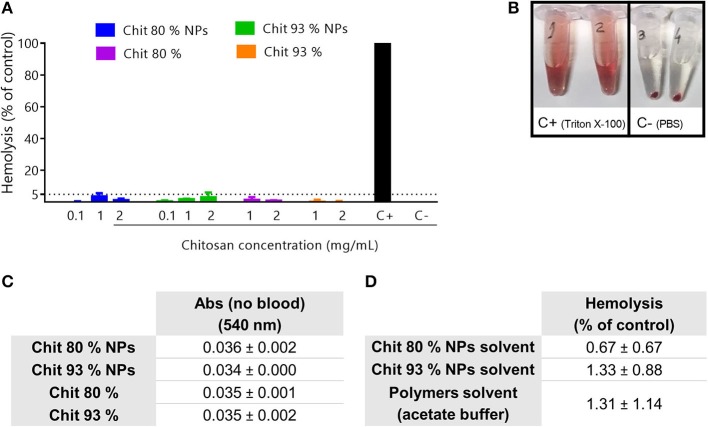
Hemolysis assay. **(A)** Hemolytic activity of Chit polymers and NPs in human blood after 3 h incubation at 37°C. PBS and Triton-X-100 were respectively used as negative (C–) and positive control (C+). Results are expressed as mean ± SEM, obtained from at least three independent experiments, using blood from different donors, each performed in duplicate (*n* ≥ 3). **(B)** Representation of 100% hemolysis generated by the positive control (tube 1 and 2) and the absence of hemolysis induced by the negative control (tube 3 and 4). **(C)** Evaluation of the Chit NP and Chit polymers interferences with the absorbance readout, without blood. Results are expressed as mean ± SEM, obtained from at least three independent experiments, using blood from different donors, each performed in duplicate (*n* ≥ 3). **(D)** Evaluation of the hemolysis resultant from the incubation of NPs solvent and polymers solvent in human blood after 3 h incubation at 37°C.

Chit NPs and polymers hemolytic activity was evaluated following a 3 h incubation at 37°C with RBCs. Results showed that none of them induced a percentage of hemolysis superior to 5%, even in Chit concentrations of 2 mg/mL (Figure 7A). Triton X-100 was used as the hemolytic agent whose effect is possible to observe by the red color of the supernatant after centrifugation of the experiment tube 1 and 2 (Figure 7B). According to the ASTM E2524-08 standard, only hemolysis superior to 5% are considered significant. Although no hemolytic activity was induced by Chit NPs and polymers, solvents were tested as well as the NP interference with the assay readout. As depicted in Figure 7C, the NPs had no interference in the absorbance measurements and Figure 7D illustrates that no hemolysis was induced by the NPs or solvents of the suspensions of the NPs or polymers.

#### The Effect of Chitosan Nanoparticles in Coagulation and Platelet Aggregation Depends on the Nanoparticle Characteristics

**Figure 8:**
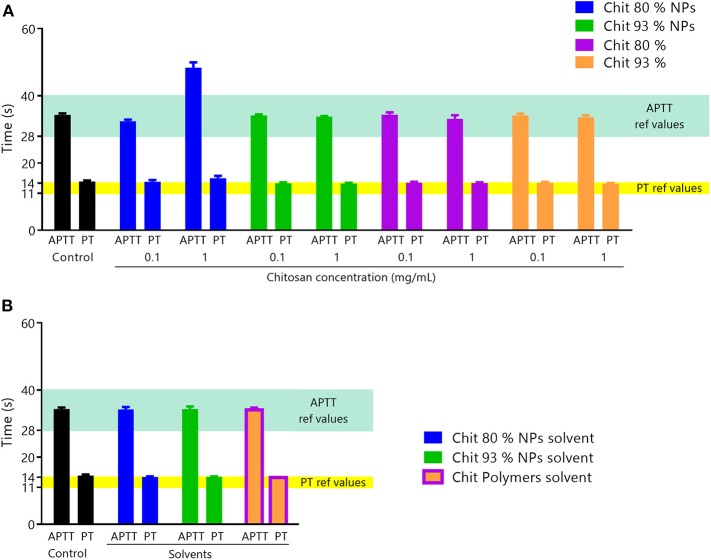
Coagulation assay. **(A)** Effect of Chit NPs and polymers at 0.1 and 1 mg/mL on plasma coagulation time after incubation for 30 min. The two coagulation pathways, APTT and PT, were separately tested. APTT reference range of values is 20–40 s and for PT is 11–14 s. Results are expressed as mean ± SEM, obtained from three independent experiments, using blood from different donors, each performed in duplicate (*n* ≥ 3). **(B)** Controls of interferences of NPs and polymers solvents with the coagulation times assay. Results are expressed as mean ± SEM, obtained from three independent experiments, using blood from different donors, each performed in duplicate (*n* ≥ 3).

The plasma coagulation cascade is responsible for blood clotting and consists of a series of protein interactions (Laloy et al., 2014). To evaluate the effect of Chit NPs and polymer samples on plasma coagulation time, two concentrations (0.1 and 1 mg/mL) of test samples were incubated with blood during 30 min. In this assay, both blood coagulation pathways, the activated partial thromboplastin time (APTT) and the prothrombin time (PT) were separately tested (Figure 8A).

The results showed that Chit NPs and polymers at 0.1 mg/mL concentration had no effect on plasma coagulation for both pathways. However, 1 mg/mL Chit 80% NPs prolonged APTT (intrinsic pathway), while no effect was observed with Chit 93% NPs and polymers 80% and 93% at the same concentration. NPs suspension solvent and polymer suspension solvent (acetate buffer) was also tested to discard any method interference and no effect was observed in plasma coagulation (Figure 8B).

Platelets play an important role not only in hemostasis but also in immune and inflammatory responses (Golebiewska and Poole, 2015). Homeostatic imbalance as a result of platelet function alterations affect primary hemostasis and can result in thrombotic or hemorrhagic disorders (Golebiewska and Poole, 2015). Therefore, it is important to study Chit NPs interactions with platelet function.

**Figure 9:**
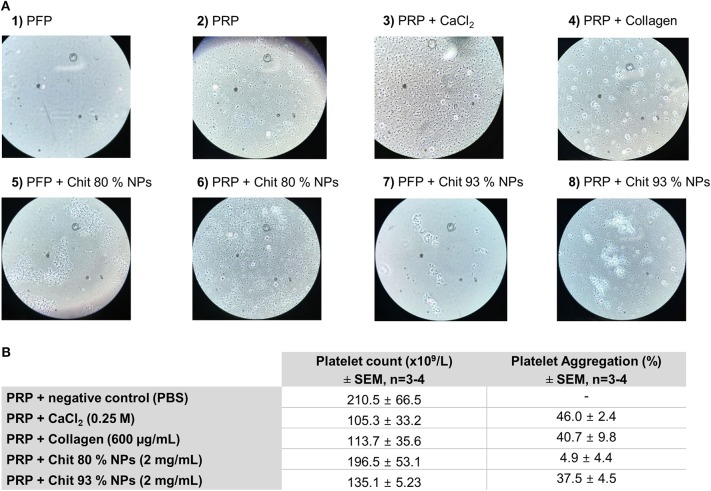
Effect of Chit 80% NPs and Chit 93% NPs in platelet aggregation. Platelet aggregation was detected by incubating PRP with 2 mg/mL of NPs for 15 min. PBS, collagen (200 and 600 μg/mL) and calcium chloride (CaCl2, 0.25 M) were used as negative and positive control, respectively. **(A)** Representative images of platelet aggregation assay stained with Giemsa dye. Untreated platelet free plasma (PFP) is represented in image 1 and untreated platelet rich plasma (PRP) is represented in image 2. For the experiment two different positive controls were used (CaCl2–3 and collagen−4). Chit 80% NPs and Chit 93% NPs were tested both with PFP (image 5 and 7) and PRP (image 6 and 8). **(B)** Quantification of the platelet aggregation effect. Platelet count is presented as the final average of a minimum of three donors ± SD. The percentage of aggregation was calculated using as reference the platelet count of the negative control, and is presented as the average of all assays ± SD (*n* ≥ 3).

To assess platelet aggregation, a cytometer is frequently used to count the platelets, however, by this method the interference of NPs, due to their size have to be taken into account. To evaluate the interference of Chit NPs with the platelet count, Chit NPs were incubated with platelet-free plasma (PFP) and visualized under the light microscope. Results showed that Chit NPs, most likely in the form of aggregates, were possibly counted as platelets, which invalidated the use of such method. To overcome this setback and assess platelet aggregation, the experiment was performed by counting platelets manually under a microscope, using a Neubauer chamber. Results from microscopy observation were summarized in Figures 9A,B.

The Figure 9A-1 clearly shows the absence of platelets typical from PFP, while plenty of platelets were observed in PRP, with no signs of aggregates (Figure 9A-2). When platelets were incubated with calcium chloride, we observed the formation of fibrins, a sign of platelet aggregation (Figure 9A-3). Similarly, collagen also induced platelet aggregation, but in this case no fibrins were observed (Figure 9A-4). When analyzing both types of Chit NPs incubated with PFP we can see their tendency to form NPs aggregates, which were hypothetically the cause of the observed interference in the cytometry technique (Figures 9A-5,7). Nevertheless, under microscopic observation, these aggregates were not misinterpreted as platelets. The Chit 80% NPs (Figure 9A-6) when incubated with PRP did not seem to induce platelet aggregation, as there was no evidence of platelet aggregates as found in the positive controls. On the other side, when Chit 93% NPs were incubated with PRP (Figure 9A-8) we observed that large NP agglomerates appear to have retained some platelets. Besides that, platelet aggregation was observed.

Using platelet count to calculate the percentage of platelet aggregation, positive controls induced an effect superior to 40%. The Chit 80% NPs did not induce platelet aggregation as only 4.9% of platelet aggregation was calculated for these samples. However, Chit 93% NPs resulted in 37.5% platelet aggregation similar to what was achieved with calcium chloride and collagen positive controls.

## Discussion

A hot topic in the nanomedicine field are polymeric NPs, which are engineered to either interact or not with the immune system. In the early stages of the development of a nanotechnology-based medicine, when the drug is to be encapsulated into NPs, the first question to be considered is, whether it is supposed that the new nanomedicine, in addition to its main pharmacological action, also acts on the immune system. This kind of approach is part of the SbD. Particularly, in the case of chitosan, as it is a set of polymers with different MW and DDA [quality attributes (QA)], it is important to understand if there are differences between them, regarding possible interactions with the immune system. For Chit NPs, in addition to polymer QA, NP characteristics, like size and zeta potential or shape can also be important. Therefore, physicochemical characteristics (PCC) of the polymers and NP might influence their immunological properties, and therefore a thorough characterization of both is very important to supplement the immunotoxicity studies and to draw meaningful conclusions (Crist et al., 2013). The lack of an exhaustive characterization may preclude the correct interpretation of results and may lead to misinterpretations hindering the establishment of trends regarding how Chit NP PCC influence the immune response. Additionally, one of the most important challenges encountered in *in vitro* immunotoxicity tests for NPs is related to their unique physicochemical properties. These can interfere with the established tests, originally developed for testing conventional chemicals (Dobrovolskaia and McNeil, 2016). Such interference depends both, on the NPs tested and the *in vitro* assay and can lead to false-positive or false-negative results (Dobrovolskaia and McNeil, 2016). Lastly, in order to achieve a correct result interpretation, it is important to identify the presence of biological contaminants in the NP preparation (Dobrovolskaia and McNeil, 2007). The main biological contamination in *in vitro* assays, even when working under sterile conditions, are endotoxins, which may lead to inflammatory responses (Dobrovolskaia and McNeil, 2007).

The present case study intends to provide a systematic analysis of the effects of Chit NPs and respective Chit polymers on different biological outcomes commonly tested under the immunotoxicity scope, considering, as most important the effect of DDA and MW, without neglecting possible interferences and contaminants.

In detail, as literature suggests, we found that Chit NPs appear to be more cytotoxic than the respective Chit polymers from which they were derived. In fact, for polymer concentrations up to the extraordinary concentration of 5,000 μg/mL, no cytotoxic effects were found neither in PBMCs, nor in RAW 264.7 cells. On the other hand, when the polymers were assembled into NPs, the same range of Chit concentrations induced a concentration dependent reduction in cell viability. Another important result we found was that PBMCs isolated from human blood were more sensitive to the NPs than RAW 264.7 cells, which is evident from the lower IC50 values extrapolated. Furthermore, this higher sensitivity of PBMCs exposed differences between the NPs produced with Chit 80% and Chit 93%. In fact, Chit 80% NPs induced a more accentuated decrease in cell viability. To discuss these results some aspects must be analyzed. To begin with, the cell culture media were different for PBMCs and RAW 264.7 cells (RPMI and DMEM, respectively). The physicochemical characterization of the NPs in water (stock suspension) is important, but their characterization when dispersed in the medium used for *in vitro* assays can provide further evidence. In fact, Chit 80% NPs presented a smaller size than Chit 93% NPs in water (127 nm vs. 292 nm), but these differences were not observed in cell culture media. Moreover, the NPs size analysis in cell culture media resulted in very high PDI. We realized that in RPMI (used for PBMCs) Chit 80% NPs presented an important size population around 500–1,000 nm, while Chit 93% showed a significant size population around 1,000–2,000 nm. On the other hand, Chit 80% NPs and Chit 93% NPs in DMEM (used for RAW 264.7) did not show such size distribution profile, with the most expressive populations around 300–700 nm and 400–800 nm, respectively. Therefore, the most noteworthy size differences occurred in RPMI, which could explain the different cell viability profile between the NPs in PBMCs.

Literature review showed several contradictory results regarding Chit NPs effect on cellular ROS production. One study suggested that Chit NPs had an inhibitory activity (Bor et al., 2016), two studies reported no Chit NPs effect (Omar Zaki et al., 2015; Arora et al., 2016) and three reported a stimulating effect (Hu et al., 2011; Sarangapani et al., 2018; Wang et al., 2018) on basal ROS cellular production. Concerning the polymer, same conflicting results were also found (Arora et al., 2016; Salehi et al., 2017; Sarangapani et al., 2018). From our case study, we concluded that, despite no significant differences were found in the cytotoxic profile of both NPs in RAW 264.7 cells, in ROS assay these NPs had different effects when tested at non-cytotoxic concentrations. Only Chit 80% NPs induced ROS production in a concentration-dependent manner (starting at 156 μg/mL). Nevertheless, the 80% DDA polymer suspended in acetate buffer also induced ROS production. Thus, the effect was dependent on the type of Chit polymer: Chit with the lowest DDA induced ROS production. On the other hand, neither NPs nor polymers, irrespective of the DD were able to inhibit ROS production. While our results suggested an influence of the DDA of the polymer in cellular ROS stimulation, the above mentioned studies did not. In fact, all authors mentioned used similar DDA Chit (75–85%) and no pattern could be observed. Moreover, those results are also affected by other variables, such as the different cellular models and testing conditions, namely concentrations, used by each author, as previously reviewed elsewhere (Jesus et al., 2019). Furthermore, none of the studies mentioned used RAW 264.7 cells, which hinders the comparison with the results herein presented.

Concerning the ability to induce NO by cells, only one result was found in the literature that claim the ability of the chitosan NPs to induce cells to produce this inflammatory marker and it showed a concentration-dependent increase above 68.18 μg/mL, in PBMCs following 24 h incubation (Pattani et al., 2009). Our case study, however, did not allow us to confirm this trend. Our results showed that none of the Chit NP tested increased NO production, in the range 39 μg/mL to 156 μg/mL. To note, Pattani et al. used Chit NPs cross-linked with sodium carboxymethyl cellulose that possessed a much smaller average size (37 nm), which may have been one of the causes for the increased reactivity. For Chit polymer, two studies observed no effect in basal NO production (Jeong et al., 2000; Wu and Tsai, 2007) supporting our results (Chit polymers did not induce NO production), while two others reported an increase (Peluso et al., 1994; Wu et al., 2015). In the case of Peluso et al. (1994) we can hypothesize that the conflicting results can be due to the use of a different cellular model (rat peritoneal exudate macrophages) or a possible endotoxin contamination, which was not assessed. On the other hand, Wu et al. (2015) used RAW 264.7 cells and claimed the endotoxin level in the stock solution was <0.5 EU/mL, which is a much higher value than we have for the Chit polymers tested. In opposition, the ability of Chit NPs and polymers to inhibit LPS-induced NO production was verified for all testing samples. This effect was similar among them, suggesting no effect of the DDA or particle size. In this case, although we have excluded that Chit NPs or polymers were interacting with NO, hampering its quantification, we cannot rule out the ability of Chit to bind LPS, partially inhibiting its effect. These findings of NO inhibition are in agreement with most of the results found in the literature, where Chit NPs were reported to inhibit H_2_O_2_-stimulated NO production (Wen et al., 2013) and Chit was reported to inhibit LPS-induced NO production (Hwang et al., 2000; Wu and Tsai, 2007). In contrast, one study performed by Jeong et al. showed Chit had a synergistic effect with IFN-γ to induce NO production (Jeong et al., 2000). In this case, the polymer used had a higher MW (300 KDa) than the polymer used in this study.

Regarding the ability of the Chit polymer and NPs to stimulate cell to produce pro-inflammatory cytokines, for instance the induction of TNF-α has been reported in some studies. These studies, however, must be carefully discussed regarding endotoxin contamination. We realized that when the authors do not disclose the purity of the polymer used, namely whether it is an LPS-free chitosan or not, results are not consensual. In some of these studies, IL-6 and TNF-α were reported to be induced following Chit and Chit NPs stimulation (Feng et al., 2004; Koppolu and Zaharoff, 2013; Baram et al., 2014), while in others studies they were not (Villiers et al., 2009; Han et al., 2016). On the other hand, when authors used Chit-based samples prepared under endotoxin-free conditions (Pattani et al., 2009; Lieder et al., 2013; Stopinšek et al., 2016), they were unanimous proving that “pure/clean” non-cytotoxic Chit and particularly, Chit NPs, do not induce IL-6 or TNF-α secretion. In agreement with this, our endotoxin-free formulations confirmed that Chit NPs and polymers do not induce TNF-α or IL-6 secretion in PBMCs. Consistently, previous studies from our group using different endotoxin-free Chit-based particles (different DDA and MW polymer, cross-link compound and NPs size when compared with present NPs) also showed no ability to induce these cytokines in mice spleen cells (Soares et al., 2019) and mice bone marrow derived dendritic cells (BMDCs) (Lebre et al., 2019). However, the last study (Lebre et al., 2019) proved that Chit and Chit NPs were able to stimulate BMDCs, activating the NLRP3 inflammasome. As a consequence, it was observed an increase of the IL-1β (pro-inflammatory cytokine) secretion by cells.

Regarding hemocompatibility assessment, our studies also allowed to clarify some conflicting literature results. Considering the hemolytic activity, some original articles were found supporting the non-hemolytic activity of Chit NPs (Nadesh et al., 2013; Kumar et al., 2017) and also the Chit polymer (Nadesh et al., 2013). Nevertheless, two studies reported a slight hemolytic effect for Chit NPs (Shelma and Sharma, 2011; de Lima et al., 2015). The last study, however, suggested that the hemolytic activity was due to the NPs solvent, which was diluted acetic acid and neutralized diluted acetic acid (de Lima et al., 2015). Our studies enabled us to confirm that both Chit polymers and Chit NPs do not have hemolytic activity even at high concentrations (2 mg/mL) and that the washing procedure of the NPs eliminated the acetic acid traces of the NPs solvent, which could otherwise induce an erroneous hemolysis. Concerning coagulation studies, we found that only Chit 80% NPs caused a concentration dependent effect on coagulation. At similar concentrations, the Chit 93% NPs and both Chit polymers in acetate buffer had no effect, meaning the effect was dependent on the nanoscale dimension of the NPs and on the polymer characteristics (80% DDA and 49 kDa). We can hypothesize that Chit 80% NPs prolonged activated partial thromboplastin time due to the affinity of NPs for plasma clotting factors that are involved in the intrinsic pathway (XII, XI, IX, VIII), possibly adsorbing them (Palta et al., 2014). In previous studies, Shelma and Sharma (2011) showed that Chit NPs reduced the total normal coagulation time, while Nadesh et al. verified that Chit NPs did not alter coagulation time, when resuspended in saline (Nadesh et al., 2013). However, experimental conditions were significantly different. The first used 2 mg/mL which is a concentration similar to ours, but evaluated only the blood clotting time, and the second only used 0.05 mg/mL. Lastly, only Chit 93% NPs were able to induce platelet aggregation. We can hypothesize this effect was only observed with Chit 93% NPs due to the higher amount of ${\text{NH}}_{3}^{+}$ groups resulting from deacetylation, increasing the interaction with negatively charged groups of platelets. However, through microscope slide analysis we postulated that the effect may also be related to the formation of large NP aggregates when using a concentration of 2 mg/mL, that further leads to platelet aggregation at their surface. Accordingly, Shelma and Sharma (2011) also reported that platelet aggregation was induced by Chit NPs at a concentration of 2 mg/mL. However, since in the same Chit 80% NPs concentration we could not confirm this tendency, the influence of different physicochemical properties of NPs affecting the biological activity must be highlighted.

In addition to the specific immunotoxicity and hemocompatibility results presented here, this case study aims to raise awareness of the scientific community about the importance of adequate controls (experimental and sample controls). Indeed, some studies fail to report important experimental controls to validate whether a particular assay is appropriate for each NP formulation and to avoid false-positive and false-negative results. A simple control is the evaluation of NP interference in the assay readout (absorbance, luminescence or fluorescence) in the absence of the biological matrix. This is omitted most of the times even though it highly increases the reliability of the obtained results. For instance, in the platelet aggregation study, the cytometer counted NPs instead of platelets, which was the reason why we did not use this technique and we had to use a light microscope. Another desirable experimental control is the cellular viability at the end of each assay, to guarantee that the revealed effects are not only a side effect of cytotoxic concentrations. Regarding sample controls, a parameter that is generally ignored is the solvent of the NP suspension. Usually, synthesized nanoparticles are in a solvent which is not designed to be biocompatible, but to stabilize the particles and prevent their aggregation in stock suspensions. The presence of such solvent in the culture medium may be enough to induce cell death, alter osmolality, pH, cause cellular damage, and decrease metabolic activity (Oostingh et al., 2011). Therefore, solvent control test is also useful to correctly interpret the results. Ultimately, we highlight the need to avoid endotoxin contamination of polymeric NPs, as it is frequently not considered and may be the source of false bioactivity or toxicity assumptions. In fact, endotoxins are a type of bacterial cell wall toxins, responsible for inducing a state of inflammation in organisms, resulting in fever, fibril reactions and organ damage (Dobrovolskaia et al., 2009). NPs are typically contaminated with endotoxins, mostly Chit NPs as their marked positive surface charge is especially susceptible to this kind of contamination. We believe that the increasing awareness of researchers about endotoxin contamination will contribute to reduce the disparity among NP immunotoxicity results.

Once more, we confirmed that our Chit NPs are more cytotoxic than Chit polymers, which justifies why we cannot rely on the Chit polymer attested safety to extrapolate to Chit NPs. More importantly, as we proposed, the presented results enabled us to shed light on some conflicting results found in literature. Notably, neither Chit NPs tested here demonstrated intrinsic pro-inflammatory ability. However, other assays showed that Chit DDA and MW influence Chit NPs immunotoxicity and hemocompatibility. Chit NPs with the lower DDA and lower MW (Chit 80% NPs) were more toxic in terms of reducing cell viability, ROS production and coagulation times. Nevertheless, all reported effects are concentration dependent and do not refrain Chit 80% NPs from being promising drug delivery systems or vaccine adjuvants.

To conclude, the present case-study together with further studies may contribute to the development of a knowledge-based guideline that enables NP product design based on the SbD approach. Nevertheless, we cannot overlook the current need to establish a set of methods for immunotoxicological assessments of NPs that need validation and standardization to allow the generation of a reliable database of results, essential to apply SbD more efficiently.

## Data Availability Statement

All datasets generated for this study are included in the article/Supplementary Material.

## Author Contributions

SJ, AM, and OB contributed conception and design of the study. AM, AD, ES, JC, and MC performed the experimental work. SJ wrote the first draft of the manuscript. SJ, AM, ES, MS, CS, GB, PW, and OB contributed to data analysis and manuscript revision. All authors read and approved the submitted version.

### Conflict of Interest

The authors declare that the research was conducted in the absence of any commercial or financial relationships that could be construed as a potential conflict of interest.
