# Supplementary material for: Chitosan Nanoparticles: Shedding Light on Immunotoxicity and Hemocompatibility
Source: Front Bioeng Biotechnol. 2020 Feb 21;8:100. doi: 10.3389/fbioe.2020.00100 (PMC7047933; doi:10.3389/fbioe.2020.00100)
Supplement: Supplementary file 1 [file Table_1.DOCX]

Supplementary Material

**Supplementary Figure S1.** Cell viability assessment after immunotoxicity assays with RAW 264.7 cell line. A) MTT assay after ROS production assay. B) MTT assay after inhibition of ROS production assay. C) MTT assay after NO production assay. D) MTT assay after inhibition of NO production assay.
